# The online application of binding condition B in native and non-native pronoun resolution

**DOI:** 10.3389/fpsyg.2014.00147

**Published:** 2014-02-25

**Authors:** Clare Patterson, Helena Trompelt, Claudia Felser

**Affiliations:** Potsdam Research Institute for Multilingualism, Faculty of Human Sciences, University of PotsdamPotsdam, Germany

**Keywords:** pronoun resolution, binding, sentence processing, eye-movement monitoring, bilingualism, English

## Abstract

Previous research has shown that anaphor resolution in a non-native language may be more vulnerable to interference from structurally inappropriate antecedents compared to native anaphor resolution. To test whether previous findings on reflexive anaphors generalize to non-reflexive pronouns, we carried out an eye-movement monitoring study investigating the application of binding condition B during native and non-native sentence processing. In two online reading experiments we examined when during processing local and/or non-local antecedents for pronouns were considered in different types of syntactic environment. Our results demonstrate that both native English speakers and native German-speaking learners of English showed online sensitivity to binding condition B in that they did not consider syntactically inappropriate antecedents. For pronouns thought to be exempt from condition B (so-called “short-distance pronouns”), the native readers showed a weak preference for the local antecedent during processing. The non-native readers, on the other hand, showed a preference for the matrix subject even where local coreference was permitted, and despite demonstrating awareness of short-distance pronouns' referential ambiguity in a complementary offline task. This indicates that non-native comprehenders are less sensitive during processing to structural cues that render pronouns exempt from condition B, and prefer to link a pronoun to a salient subject antecedent instead.

## Introduction

During language comprehension linguistic structure must be encoded, and rapid decisions about dependency formation such as pronominal reference need to be made. Whilst it is generally agreed that processing a pronoun involves the retrieval or reactivation of an antecedent (either explicit or understood from the context), there is no clear consensus on the precise role that structural constraints play in this retrieval process.

Much of the recent debate in this area has been around the memory processes involved in long-distance dependencies, with particular reference to reflexive processing and subject-verb agreement (see Dillon, 2011, for an overview). One view is that reflexive processing in particular involves a structure-sensitive search, so that the target of the retrieval is identified through its position in the linguistic structure (Dillon, 2011; Dillon et al., 2013). An opposing view is that retrieval for reflexives exploits the cues carried on prior representations, so that, for example, a singular, masculine reflexive triggers a search for representations carrying the features *singular* and *masculine*. Importantly, this second approach predicts that retrieval interference is possible from antecedents that are not structurally licensed (e.g., Patil, 2012).

As far as pronouns^1^ are concerned, structure alone is not sufficient to uniquely identify a referent, and the interpretation of pronouns is subject not only to structural constraints but also a range of discourse constraints, distinguishing it from reflexive interpretation. Despite this, there is debate around the primacy of the structure-sensitive constraint known as condition B of the Binding Theory (Chomsky, 1981). Condition B restricts the interpretation of pronouns such that a pronoun cannot refer to a c-commanding antecedent within its local binding domain^2^. For example in (1), the direct object pronoun *him* cannot refer to *David* but it can refer to *Nick*. The embedded subject *David* is “inaccessible” as a binder for *him* because the two are coarguments of the same predicate.

(1) Nick_*i*_ thinks that David_*k*_ likes him_*i*,^*^k_

Whether or not condition B can be defined in purely structural terms, though, is debatable. Binding Theory assumes an exclusion on the basis of structural position, but other views involve excluding the inaccessible antecedent on mainly pragmatic grounds (Huang, 1994) or by comparing two alternative semantic sentence representations (Reinhart, 1983; Reuland, 2001, 2011). In this paper, the term “condition B” will henceforth be used as a general term to express the exclusion of inaccessible antecedents for pronouns, rather than endorsing a particular theoretical approach.

According to the *binding as initial filter* (BAIF) hypothesis by Nicol and Swinney (1989), condition B is used to exclude inaccessible antecedents from an early stage of processing. In the case of canonical condition B environments exemplified in (1), the local (inaccessible) antecedent would be immediately ruled out and would not influence the parse at any point. That is, condition B should prevent consideration of inaccessible antecedents even when they carry number or gender features that match those of the pronoun. Evidence for this hypothesis came from several cross-modal priming studies which found antecedent reactivation effects only for accessible but not for inaccessible antecedents (Nicol and Swinney, 1989). Further support for this hypothesis mainly comes from negative evidence in self-paced reading studies, i.e., a lack of a demonstrable effect from manipulating the gender or number features of an inaccessible antecedent. When no effect is found, the assumption is that the inaccessible antecedent is not being considered. Negative evidence of this kind has been found by Clifton et al. (1997, 1999).

A variant of the BAIF hypothesis is the idea that binding constraints may act as defeasible filters, with inaccessible antecedents potentially being considered at later processing stages. Evidence in support of this comes from an eye-movement study on English reflexives reported by Sturt (2003).

An alternative to both the BAIF and the defeasible filter hypotheses was put forward by Badecker and Straub (2002). They suggested that multiple cues or constraints that are relevant for pronoun processing (including structural constraints) all contribute in parallel, positively or negatively, to an antecedent's activation. Thus, positive activation from one constraint may be canceled out by inhibition from another. Due to this parallel activation/inhibition, the feature match or mismatch of an inaccessible antecedent will have an influence on processing, in direct contrast to the BAIF hypothesis. Badecker and Straub found that the reading times in regions following a pronoun were longer when both the accessible and inaccessible antecedents matched in gender with the pronoun, compared to when only the accessible antecedent matched. They suggested that all feature-matching referents, whether accessible or inaccessible according to Binding Theory, are evaluated. Further evidence that the inaccessible antecedent is not immediately excluded from consideration comes from Clackson et al.'s (2011) eyetracking-during-listening study. Adult participants' eye gaze patterns revealed that they experienced interference from a gender-matching but structurally inaccessible antecedent after encountering a pronoun. Such evidence can be characterized as supporting a feature-based antecedent search as proposed by Badecker and Straub.

Thus the current evidence bearing on the BAIF with respect to pronouns appears to point in two directions, and there is as yet no clear consensus on whether or not condition B gates access to certain potential antecedents during processing.

In order to establish a broader picture of the mechanisms behind pronoun processing, environments which are exempt from condition B should also be considered. While there are, of course, many syntactic environments in which condition B plays no role (because there is no inaccessible antecedent to exclude) the use of specific exceptions to condition B is more informative. In these cases, condition B *should* apply to rule out a local antecedent, but it does not. The exception that is made use of in the current study is the case of so-called “short distance pronouns” (SDPs). In certain structures such as (2) below, a local c-commanding noun phrase (NP) *can* be interpreted as the antecedent for the pronoun, and it seems that both reflexives and pronouns can appear in these positions (Lees and Klima, 1963, among others).

(2) Nick_*i*_ saw David_*k*_ put the cat beside him_*i/k*_.

Possible reasons as to why SDPs seem exempt from condition B include proposals to the effect that prepositional phrases such as *beside him* in (2), or certain kinds of (verb phrase internal) aspectual phrases, can be binding domains (Hestvik, 1991; Tenny, 2004). Under this view, the local subject *David* in (2) is outside the pronoun's binding domain and is thus allowed to bind it without condition B being violated. More widely accepted is the proposal that the scope of condition B should be restricted to anaphoric dependencies involving coarguments (e.g., Reinhart and Reuland, 1993). This also allows for the pronoun *him* in (2) to enter into a referential dependency with the local subject *David* because the two are not in fact arguments of the same predicate. Alternatively, Rooryck and Vanden Wyngaerd (2011) have proposed that rather than being bound by the local subject NP, SDPs are variable-bound by a covert operator located at the left clausal periphery. Regardless of which of the above theoretical accounts is ultimately deemed preferable, recognizing syntactic environments in which local coreference is permitted requires sensitivity to the relevant structural differences between standard condition B environments such as (1) above and SDP environments such as (2).

Exceptions such as SDPs, then, make a good comparison point with canonical condition B environments because their structure is quite similar, but they can reveal how pronoun processing unfolds when condition B appears not to apply. This may, for example, shed further light on possible feature-driven processes, or reveal an underlying sensitivity to the linear ordering of antecedents, as has been found in certain syntactic environments (Cunnings et al., 2014). The online processing of pronouns in SDP environments has rarely been investigated. Experimental evidence for the referential ambiguity of SDPs has been reported by Sekerina et al. (2004). Using eyetracking-during-listening, they examined English-speaking children and adults' processing of questions such as (3) below.

(3) Which picture shows that the boy has placed the box behind himself/him?

Participants had to choose between two alternative pictures, one of which showed the box being located behind a boy (= the sentence-internal referent) and one in which it was located behind an adult male character (= the sentence-external referent). Participants' eye-gaze patterns showed a reduced proportion of looks to the picture corresponding to sentence-internal reference resolution in the pronoun compared to the reflexive condition, suggesting that the alternative, sentence-external antecedent was more likely to be considered in the pronoun than in the reflexive condition. In a corresponding offline task, the adult participants showed a strong across-the-board preference for sentence-internal antecedents. The focus of Sekerina et al.'s study was on sentence internal vs. external antecedents, and possible differences between antecedent preferences for reflexives vs. pronouns. It does not give a broader picture of pronoun processing in environments with two potential sentence-internal antecedents, although it is interesting to note that pronouns appear to be more flexible in their interpretation than reflexives. In our current study, we use SDP environments such as (2) as a contrast to condition B environments. The crucial factor here is that both antecedents are thought to be accessible to the pronoun.

There are other environments which appear to be exempt from condition B; so-called “picture noun phrases” are a well-studied example (Runner et al., 2003; Kaiser et al., 2009, among others)^3^. The main finding from these studies regarding pronouns is that non-structural factors such as semantic role information are important. Most relevant to the current study, however, is that previous studies have shown that native English-speaking comprehenders are aware of the referential ambiguity of binding-theory exempt pronouns during processing.

### Non-native processing of pronominal anaphors

It is not only exceptions to condition B that can provide a broader picture about the processing of pronouns. The processing profiles of different populations, in this case non-native speakers, can also be informative. Models of parsing, particularly those that are closely tied to aspects of general cognition, should be able to account not only for native language processing but also for processing in a non-native language. Additionally, non-native speakers have been shown in previous studies to take a more discourse-driven strategy than native speakers during the processing of, for example, reflexives (Felser and Cunnings, 2012), findings which appear to challenge the universal validity of serial or syntax-first models that were proposed on the basis of monolingual processing data.

Most previous research on non-native anaphor resolution has examined learners' knowledge of binding using offline judgment or antecedent choice tasks. Unlike the developmental delay of condition B that has been reported in the child language acquisition literature (e.g., Chien and Wexler, 1990), the application of binding condition B appears to be relatively unproblematic in the post-childhood acquisition of non-native speakers (henceforth L2s). White (1998), for example, reports that even intermediate-level L2 learners of English patterned with English native speakers in a truth-value judgment task in disallowing local antecedents for pronouns. Using a multiple-choice antecedent identification task, Bertenshaw (2009) found that native Japanese-speaking learners of English correctly rejected inaccessible antecedents for pronouns 92.8% of the time, a figure that compares favorably with the native speaker controls' correct rejection rate of 87.5%. Similarly high accuracy rates have been reported by Cook (1990).

Conversely, little is known about whether or when binding constraints are applied during online L2 processing. L2s have been claimed to show reduced sensitivity to syntactic information during processing compared to native speakers (henceforth L1s), and difficulty establishing structurally mediated discontinuous dependencies in a native-like way (Clahsen and Felser, 2006). However, a reduced ability to process syntactically mediated dependencies may affect L2 online interpretation of reflexives more than the ability to interpret pronouns, all other things being equal. This is under the assumption that binding of argument reflexives is contingent on mechanisms of syntactic computation, whereas non-reflexive pronouns can also be linked to an antecedent via discourse-based coreference assignment (e.g., Reuland, 2001, 2011).

While L1 speakers appear to respect condition A of the Binding Theory (which states that reflexives must be locally bound) from the earliest measurable point in processing (Sturt, 2003; Xiang et al., 2009), a different picture emerges in L2 processing. Felser et al. (2009) report evidence from timed grammaticality judgments and eye-movement monitoring showing that native Japanese speakers experienced competition from inaccessible antecedents for English argument reflexives during processing, despite demonstrating native-like knowledge of binding condition A in complementary offline tasks. Felser and Cunnings (2012) further explored the interaction of structural and discourse factors in non-native anaphor resolution by examining native German speakers' processing of English reflexives. Two eye-movement monitoring experiments were carried out using sentences such as (4a) and (4b) in a gender-mismatch paradigm (compare e.g., Sturt, 2003).

(4a) James has worked at the army hospital for years. He noticed that the soldier had wounded himself while on duty in the Far East.
(4b) James has worked at the army hospital for years. The soldier that he treated on the ward wounded himself while on duty in the Far East.

The L2s' reading-time patterns differed from the L1s' in that they initially showed unmodulated main effects of the inaccessible antecedent's gender only. This was the case both for sentences like (4a), in which the inaccessible antecedent (the pronoun *he*) c-commands the reflexive, and for sentences such as (4b), where it does not. Only in later measures and/or sentence regions did the L2 speakers pattern with the L1 controls in showing main effects of the accessible antecedent's gender. Taken together, these results indicate that unlike L1s, L2 speakers do not immediately apply binding condition A during processing but initially try to link argument reflexives to the most discourse-prominent antecedent via coreference assignment instead.

To our knowledge, the timing of binding condition B during L2 pronoun processing has never been investigated. L2 processing studies on pronoun resolution have focused on discourse anaphors rather than bound pronouns. The findings from these studies suggest that L2s can use information-structural cues such as focus to guide pronoun resolution (Ellert, 2010) and may experience more competition than L1s in the presence of more than one feature-matching discourse antecedent (Roberts et al., 2008). Roberts et al. examined the role of contextual information in native Turkish and German speakers' real-time comprehension of ambiguous pronouns in L2 Dutch also using eye-movement monitoring. The two L2 groups patterned together in showing elevated total and second-pass reading times at the pronoun region when two (rather than only one) matching antecedents were present in the sentence-external discourse. The native Dutch controls, on the other hand, were not measurably distracted by the presence of another matching discourse antecedent.

Two experiments are described below which aim to explore the application and timing of condition B during L1 and L2 sentence processing using eye-movement monitoring during reading. To obtain information about participants' ultimate interpretation preferences, the two online reading experiments are complemented by an offline antecedent choice task (Experiment 1). Our first eye-movement experiment (Experiment 2) examines readers' processing of canonical condition B sentences such as (1) above, while Experiment 3 examines online pronoun resolution in SDP environments such as (2). Experiments 2 and 3 were run concurrently during the same experimental session. All experimental sentences contained one pronoun and two potential antecedents, local and non-local.

The following specific questions will be explored:

- Does condition B immediately exclude inaccessible antecedents from the candidate set?
- Does the order/timing of considering the two antecedents differ according to whether or not condition B applies?
- Are there any L1/L2 differences in the application of condition B?

We begin by reporting the results from the offline questionnaire study.

## Materials and methods, experiment 1

The purpose of Experiment 1, an offline antecedent choice task, was to examine the offline antecedent choices of L1 and L2 participants in the two different syntactic environments under investigation, in the absence of any time pressure. This is especially important for the SDPs because they are thought to be ambiguous.

### Participants

The L1 group comprised 83 participants, all of whom reported that they were native speakers of English (33 males, mean age 40 years, range 19–72 years). They were recruited via email and word of mouth to people who were known to be native speakers of English, and through an advertisement on an English-language forum on the internet. The L2 group comprised 35 native German-speaking students at the University of Potsdam (10 males, mean age 22.2, range 19–37 years) who had learned English as their second language at school^4^. All L2 speakers participated in a subpart of the grammar section of the Oxford Placement Test (OPT; Allan, 2004). Their mean score was 39/50 (*proficient*), range 30–48 (*lower intermediate* to *expert user*).

### Materials

The materials were ten sentences in which pronoun interpretation was constrained by condition B such as (5) below, and ten sentences containing SDPs such as (6).

(5) The boy remembered that Matthew had bought him a new computer game.
(6) Harry heard William pull the curtain around him in the quiet hospital ward.

The critical sentences all contained a direct object pronoun and two potential antecedents which matched the pronoun in gender. In (5), the local antecedent *Matthew* is ruled out by condition B, whereas in (6), it should be possible for the pronoun to be linked to either the non-local antecedent (*Harry*) or the local one (*William*). Within each experimental condition an equal number of masculine and feminine pronouns was used. We also took care to create scenarios in which the local and the non-local antecedent were equally plausible as antecedents for the pronoun.

The experimental sentences were mixed and pseudo-randomized with 22 filler sentences containing ambiguous or unambiguous pronouns and reflexives in different syntactic environments, yielding a total of 42 items.

### Procedure

The questionnaire was administered via the internet using SurveyGizmo (surveygizmo.com). The L1 group completed the questionnaire remotely. The L2 participants completed the questionnaire as part of the experimental session for online Experiments 2 and 3, after they had finished the online element. Because the experimenters had less direct control over the conditions in which the L1 participants did the questionnaire, a larger number of L1 participants were included to increase the reliability of the responses^5^.

All participants were instructed to read each sentence carefully and decide who the pronoun probably referred to. The use of *probably* takes account of the fact that another interpretation is possible, although unlikely. After each sentence the same question appeared: “Who does [pronoun] refer to?” In each case participants were given three choices as in (7) below.

(7) The boy remembered that Matthew had bought him a new computer game. *Who does “him” refer to?*
  - The boy
  - Matthew
  - Either

The order of the two antecedent responses was varied throughout the questionnaire, and the *either* option always appeared at the bottom.

## Results, experiment 1

**Figure 1:**
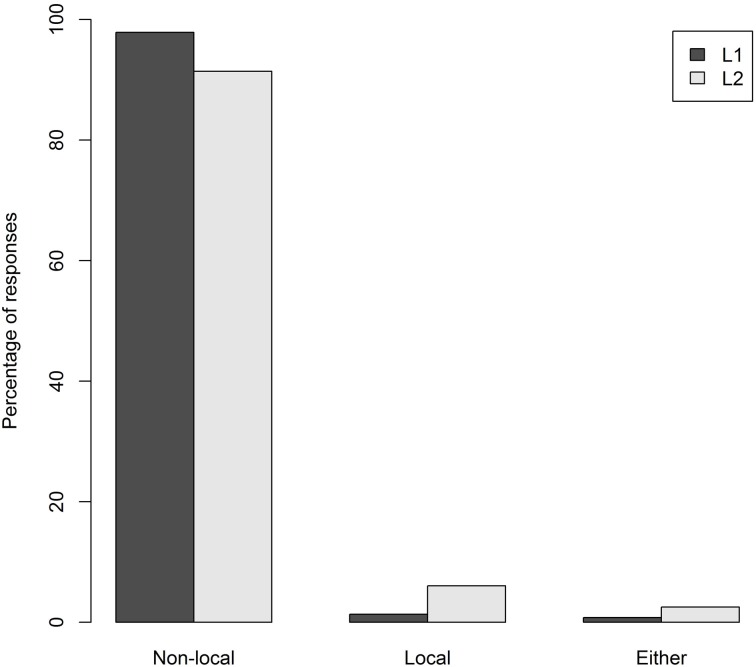
**Percentage of responses per group to the canonical condition B structures**.

**Figure 2:**
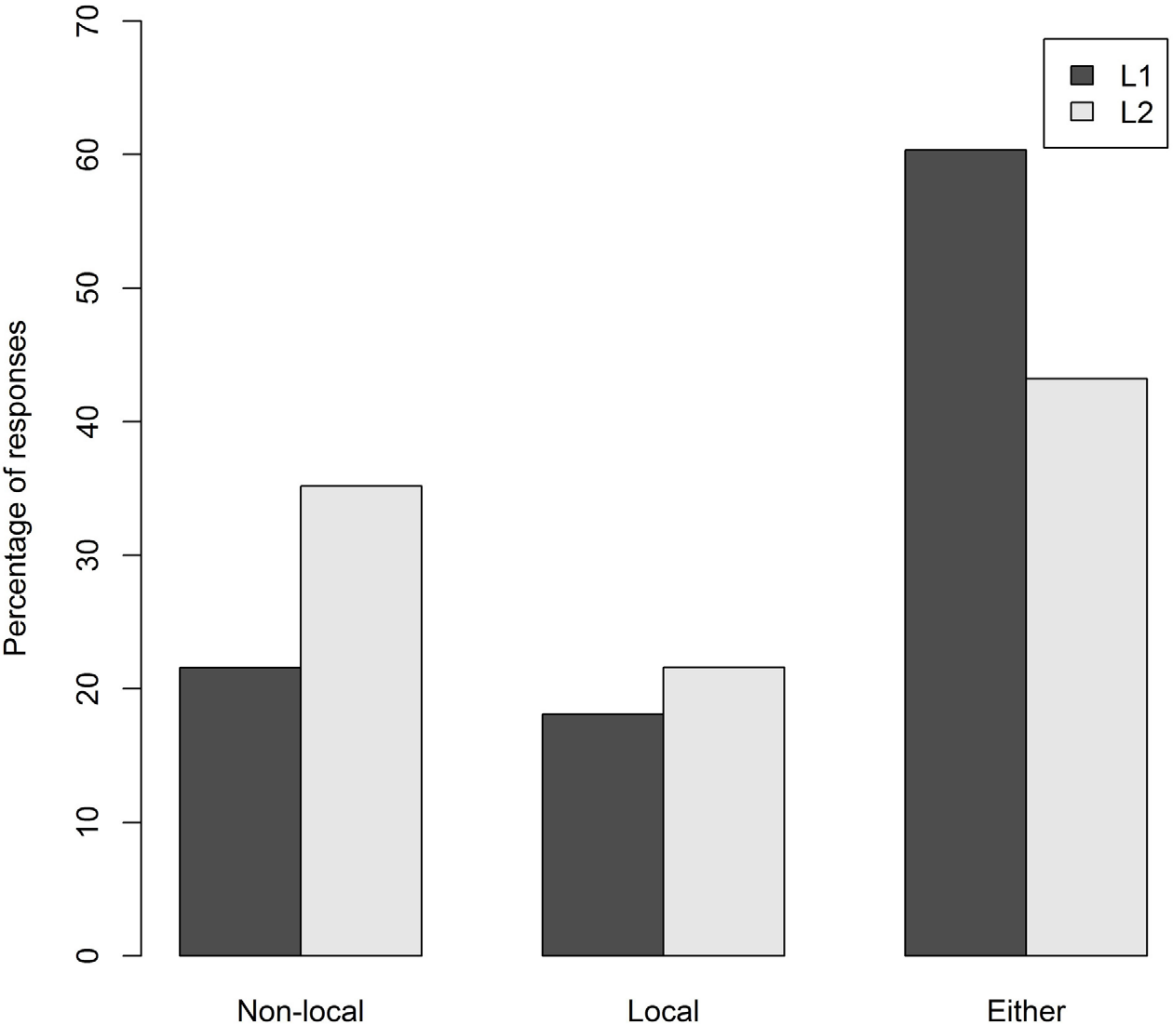
**Percentage of responses per group to the SDP structures**.

One item was removed from the analysis of the condition B sentences because it could be construed as being ambiguous. Figures 1, 2 show the percentage of responses to the canonical condition B structures and the SDP structures, for each group.

For the canonical condition B structures (Figure 1), the preference for the non-local (accessible) antecedent is very clear in both groups; they both chose this option above 90% of the time (L1 98%, L2 91%). A 3 × 2 ANOVA with an appropriate logistic transformation (Agresti, 2002) of the response rates of each type (*non-local*, *local*, and *either*) showed a main effect of antecedent choice [*F*_1(2, 232)_ = 2110.3, *p* < 0.0001; *F*_2(2, 32)_ = 349.2, *p* < 0.0001] and an interaction between antecedent choice and group [*F*_1(2, 232)_ = 19.4, *p* < 0.001; *F*_2(2, 32)_ = 49.6, *p* < 0.001]. The L1 group chose the *non-local* response more often than the L2 group [*t*_1(116)_ = 5.3, *p* < 0.001; *t*_2(16)_ = 19.8, *p* < 0.001]. Nevertheless, within-group *t*-tests confirmed that in both groups the percentage of *non-local* responses was significantly higher than that of *local* responses [L1: *t*_1(82)_ = 55.8, *p* < 0.001; *t*_2(8)_ = 20.8, *p* < 0.001; L2: *t*_1(34)_ = 18.5, *p* < 0.001; *t*_2(8)_ = 12.2, *p* < 0.001] and *either* responses [L1: *t*_1(82)_ = 68.8, *p* < 0.001; *t*_2(8)_ = 21.4, *p* < 0.001; L2:*t*_1(34)_ = 26.9, *p* < 0.001; *t*_2(8)_ = 9.1, *p* < 0.001].

Compared to the canonical condition B structures, for the SDP structures (Figure 2) there was more variability in the two groups' responses. There was a numerical preference in both groups for choosing the *either* response indicating that the pronoun was ambiguous (L1 60%; L2 43%). A 3 × 2 ANOVA showed a main effect of antecedent choice [*F*_1(2, 232)_ = 24.0, *p* < 0.0001; *F*_2(2, 36)_ = 16.7, *p* < 0.0001] and an interaction between antecedent choice and group [*F*_1(2, 232)_ = 6.6, *p* < 0.01; *F*_2(2, 36)_ = 4.1, *p* < 0.05]. For the L1 group the *either* option was chosen significantly more often than both the *local* response [*t*_1(82)_ = 7.3, *p* < 0.001; *t*_2(9)_ = 6.1, *p* < 0.001] and the *non-local* response [*t*_1(82)_ = 6.6, *p* < 0.001; *t*_2(9)_ = 4.3, *p* < 0.01]. For the L2 group the *either* response was chosen significantly more often than the *local* response [*t*_1(34)_ = 2.2, *p* < 0.05; *t*_2(9)_ = 3.9, *p* < 0.01] but not significantly more than the *non-local* response [*t*_1(34)_ = 0.4, *p* = 0.6; *t*_2(9)_ = 1.2, *p* < 0.2]. When the *either* option was not chosen, the L1 group chose the local and non-local antecedent at roughly the same rate (18 and 22% respectively); a *t*-test showed no significant difference between these two response rates [*t*_1(82)_ = 2.0, *p* = 0.5; *t*_2(9)_ = 0.3, *p* = 0.7]. The L2 group, however, chose the non-local antecedent more often than the local antecedent (34 and 21% respectively), a difference which proved (marginally) significant in a *t*-test [*t*_1(34)_ = 2.7 *p* < 0.01; *t*_2(9)_ = 2.1, *p* = 0.063]. There was a significant negative correlation between participants' OPT scores and *non-local* antecedent choice rates for the SDP structures [*r*_(35)_ = −0.35, *p* < 0.05], however, no participant categorically chose *non-local* responses.

### Experiment 1 summary

Participants' responses to the canonical condition B structures were highly consistent for both groups. While participants in the L1 group were overall more likely than those in the L2 group to choose the non-local antecedent, there was an overwhelming preference for the non-local antecedent in both groups, almost to the exclusion of any other response. This demonstrates that both L1 and L2 speakers are fully aware of the inaccessibility of the local antecedent, although the L1 group demonstrated more certainty than the L2 group. Participants' responses to the SDP structures were quite different, with the pronoun's ambiguity reflected in their antecedent choices. Both groups chose *either* at the highest rate, although the L2 group's rate of *either* responses was not significantly higher than their non-local responses. When choosing one particular antecedent (instead of the *either* option), the L1 group did not show a preference for either the local or non-local antecedent, whereas the L2 group displayed a slight preference for the non-local antecedent. This preference was related to OPT score; the lower a participant's OPT score, the more likely they were to choose the *non-local* referent. This may suggest that awareness of the ambiguity of SDPs increases with knowledge of English^6^. Taken together, the responses show that participants responded in line with condition B where appropriate, and displayed awareness of the ambiguity of SDPs.

## Materials and methods, experiment 2

Experiment 2 was designed to investigate the online application of condition B in sentences where only the local antecedent was accessible. We specifically sought to investigate whether L1 and/or L2 comprehenders would experience interference from the inaccessible antecedent at any point during processing.

### Participants

The L1 participants were 34 native speakers of English (11 males) who were recruited from the University of Essex (UK) and the surrounding community. Their mean age was 25.9 (range: 18–54), and all confirmed that English was their first language. The L2 group consisted of 34 of the 35 native German speakers who took part in Experiment 1 (10 males, mean age 22.8, range 19–37), all of whom had learned English as their second language at school starting at the age from 5 to 13 (mean: 9.6, *SD*: 1.7). Their mean OPT score was 39/50 (*proficient*), range 30–48 (*lower intermediate* to *expert user*). All participants were paid for their participation, and all had normal or corrected-to-normal vision.

### Materials

Twenty-four experimental items were constructed. They were composed of three sentences: a lead-in sentence, a critical sentence that contained the pronoun and two potential antecedent NPs that were both proper names, and a wrap-up sentence. The gender match between the two names and the pronoun was manipulated to create three experimental conditions as shown in (8a–c) below^7^.

(8) Band practice was beginning to get rather dull.
  - Double match condition
    John remembered that Mark had taught him a new song on the guitar.
  - Local mismatch condition
    John remembered that Jane had taught him a new song on the guitar.
  - Non-local mismatch condition
    Jane remembered that John had taught him a new song on the guitar.
    That really lifted everyone's spirits!

The names were matched in letter and syllable length, and were either typical male or typical female names (i.e., names that are not normally used for both genders). The names were counter-balanced across items to control for any potential frequency effects. The first name (the non-local antecedent) was always the main clause subject and was an accessible antecedent by virtue of being outside the local binding domain. The second name (the local antecedent) was always the subject of an embedded complement clause and a coargument of the pronoun. It was thus an inaccessible antecedent for the pronoun according to condition B. Half the pronouns were masculine and half feminine, and they were always object pronouns.

The experimental items were distributed across three presentation lists using a Latin-square design, and mixed and pseudo-randomized with 18 experimental items from Experiment 3 (described below) and 44 additional filler items, resulting in 86 items per list in total. The set of fillers included eight pseudo-fillers which were structurally similar to the experimental items but contained reflexive rather than non-reflexive pronouns, and another eight in which the structurally illicit antecedent for the pronoun was placed first. This was to ensure that participants were exposed to enough items that were similar to the experimental items but different in crucial factors (type of referring expression and position of the antecedent), to prevent them from developing expectations about the pronoun–antecedent relationships under investigation. Binary yes/no comprehension questions followed two thirds of the 86 items in each list, including the experimental items, to ensure that participants were paying attention and reading the items properly. A few of the comprehension questions following filler items directly probed the referent of a pronoun, to encourage participants to fully process the pronouns that they read. The experiment began with the presentation of six practice items to familiarize participants with the procedure, two of which were followed by a question.

### Predictions

In the light of the different proposals regarding the primacy of condition B during processing, the following predictions can be made.

#### BAIF hypothesis

If structural information helps to rule out inaccessible antecedents at an early point, only the accessible (non-local) antecedent should be considered. This predicts that there will be a slow-down in reading times in condition (8c) (non-local mismatch) compared to the other two conditions. In addition, because the inaccessible antecedent is excluded from consideration on structural grounds, there should be no difference between condition (8a) (double match) and (8b) (local mismatch) because participants should not be sensitive to the gender of the inaccessible antecedent.

#### Defeasible filter hypothesis

Following Sturt's (2003) results for reflexives, it is possible that binding conditions act early to include or exclude certain antecedents, but the inaccessible antecedents are considered at a later point of processing. The defeasible filter account therefore predicts longer reading times for condition (8c), followed later by effects of the inaccessible antecedent which could manifest as either longer reading times in condition (8b) or as a competition effect with differences between condition (8a) and the other two conditions.

#### Feature-match hypothesis

If condition B does not immediately overrule other cues, then processing should also be sensitive to the gender features of the inaccessible antecedent initially. Readers may only home in on the accessible (i.e., the non-local) antecedent at later processing stages or sentence regions. Following Badecker and Straub (2002), if all antecedents with matching morphosyntactic or semantic features are activated on encountering the pronoun, regardless of the structural accessibility of the antecedents, participants might experience “retrieval interference” (Gordon et al., 2001; Lewis and Vasishth, 2005; Van Dyke, 2007) indexed as increased reading times when both antecedents match the pronoun in gender (condition 8a) compared to when only a single antecedent matches (conditions 8b and 8c).

### Procedure

The experimental and filler items were pseudo-randomized such that no two experimental items appeared adjacent to each other and were spread across three presentation lists in a Latin-square design. The experiment was divided into three blocks at which point participants could take a break if required. Forward and reverse orders of each list were constructed.

All items were presented in Courier New font (size 18), and displayed across up to three lines of text onscreen. Text was displayed in black on a white background. Eye movements were recorded using the EyeLink 1000 system (SR Research Ltd) at 500 Hz. Using the desktop system, the camera was located below the screen and participants placed their heads on a chin rest that was adjusted to allow a comfortable position. The distance between the eyes and the camera was 60 cm and the distance between eyes and screen 70 cm. Viewing was binocular but only the right eye was recorded. Each experimental session began with calibration of the eye-tracker on a nine-point grid. Calibration was repeated during the session if the experimenter noticed that measurement accuracy was poor. Before each trial, the screen displayed a marker positioned above the first word of the next trial. Participants were instructed to fixate upon this marker, and press a button to view the next trial, in order to control the placement of the initial fixations.

Participants read each text silently at their normal reading rate, pressing a button on a game pad once completed and after content questions requiring a yes/no push button response. The experiment session lasted approximately 30–45 min in total for L1 speakers. For the L2 participants the experiment took about 60 min because of the additional OPT, questionnaire (Experiment 1) and vocabulary test after the experiment. The vocabulary test consisted of a checklist containing all critical vocabulary items, and the learners were asked to read through the list carefully and circle any words that they were unfamiliar with.

The research was approved by the Ethics Committee of the University of Essex (L1, March 2011) and the ethics committee of the University of Potsdam (L2, application number 37/2011). Informed consent was obtained from all participants.

### Data analysis

Reading times for four regions of text are reported: the pronoun region, which contains the pronoun and the last three letters of the preceding word; the spillover region, which contains the two words following the pronoun [e.g., *a new* in (8a–c) above]; the next two words as the prefinal region [e.g., *song on* in (8a–c) above]; and the last two words of the sentence as the final region. For the statistical analysis, all reading time measures were log-transformed [log_*e*_(*x* + 1)].

Five reading time measures will be reported for these regions. First fixation is the duration of readers' initial fixation within an interest area; first-pass reading time is the summed duration of fixations within an interest area until it is exited to either the left or the right for the first time; regression path time is the sum of all fixations on a region until this region is exited to the right; rereading time is the summed duration of all fixations in a region after it was first exited to either the left or right; and total viewing time is the summed duration of all fixations within a region. Reading times for trials in which track loss occurred, and reading times in regions which were initially skipped, were treated as missing data. For rereading time, trials in which a region was not refixated after the first-pass contributed a rereading time of zero to the calculation of averages.

Short fixations of 80 ms or below within one degree of visual arc of another fixation were automatically merged, and any other extremely short (≤80 ms) or long (>1200 ms) fixations were removed. To explore whether the two participants groups patterned differently statistically, we carried out preliminary 3 × 2 ANOVAs with the factors Condition (*double match, local mismatch, non-local mismatch*) as within-subjects factor and Group (*L1, L2*) as a between-subjects factor, for each measure and interest region. Where interactions with the factor Group were found, the data from each group were analyzed separately^8^.

## Results, experiment 2

L1 participants answered 88% of the end-of-trial comprehension questions correctly and the L2 participants 86% overall, indicating that both groups paid attention to the task and read the stimulus items for meaning. Track loss accounted for 0.2% of the L1 and 0.13% of the L2 data. Skipping rates for the four reported regions were 25, 13, 11, and 6% in the L1 group and 9, 2, 4, and 0% in the L2 group.

Summaries of participants' reading times and of the ANOVA results are provided in Tables 1, 2 respectively. Results of subsequent pairwise comparisons are summarized in Table 3.

**Table 1:** **Means (standard deviations in parentheses) for five eye-movement measures at four areas of interest in Experiment 2, for each participant group**.

	**L1**					**L2**				
	**First fixation duration**	**First-pass time**	**Regression-path time**	**Rereading time**	**Total viewing time**	**First fixation duration**	**First-pass time**	**Regression-path time**	**Rereading time**	**Total viewing time**
**PRONOUN REGION**										
Double match	212 (71)	257 (132)	347 (323)	152 (233)	409 (279)	234 (72)	355 (250)	426 (364)	135 (260)	490 (388)
Local mismatch	215 (74)	267 (152)	329 (307)	115 (211)	382 (270)	234 (85)	346 (190)	415 (266)	135 (232)	481 (317)
Non-local mismatch	209 (70)	267 (154)	370 (431)	317 (463)	584 (477)	238 (85)	366 (233)	480 (453)	247 (377)	613 (420)
**SPILLOVER REGION**										
Double match	207 (76)	256 (162)	368 (402)	146 (230)	403 (261)	241 (96)	377 (220)	439 (368)	167 (336)	544 (418)
Local mismatch	211 (71)	260 (155)	328 (311)	142 (220)	402 (270)	233 (77)	366 (183)	420 (421)	136 (284)	502 (357)
Non-local mismatch	205 (60)	260 (148)	419 (460)	250 (350)	510 (386)	254 (103)	405 (228)	589 (595)	275 (421)	680 (508)
**PREFINAL REGION**										
Double match	210 (77)	297 (164)	381 (273)	171 (253)	468 (297)	239 (88)	422 (216)	530 (401)	238 (406)	661 (464)
Local mismatch	203 (67)	295 (171)	423 (396)	163 (225)	458 (282)	240 (88)	439 (227)	485 (298)	169 (276)	608 (355)
Non-local mismatch	205 (78)	288 (162)	503 (659)	216 (314)	504 (349)	249 (95)	466 (257)	646 (629)	267 (391)	733 (457)
**FINAL REGION**										
Double match	215 (85)	311 (202)	941 (1393)	130 (259)	441 (347)	245 (94)	520 (322)	1066 (1143)	181 (363)	701 (486)
Local mismatch	224 (106)	322 (198)	846 (1054)	105 (209)	427 (299)	246 (107)	508 (326)	1014 (1194)	151 (294)	659 (421)
Non-local mismatch	212 (91)	307 (198)	1199 (1685)	124 (229)	431 (314)	251 (104)	526 (342)	1236 (1386)	240 (426)	767 (552)

**Table 2:** **Summary of analyses of variance for the pronoun, spillover, prefinal and final regions in Experiment 2**.

	**Pronoun region**		**Spillover region**		**Prefinal region**		**Final region**	
	***F*1**	***F*2**	***F*1**	***F*2**	***F*1**	***F*2**	***F*1**	***F*2**
**FIRST FIXATION DURATION**								
Group	(1, 66) 10.70^**^	(1, 23) 38.02^***^	(1, 66) 21.02^***^	(1, 23) 84.66^***^	(1, 66) 20.67^***^	(1, 23) 47.45^***^	(1, 66) 16.16^***^	(1, 23) 70.68^***^
Condition	(2, 132) 0.12	(2, 46) 0.18	(2, 132) 0.95	(2, 46) 1.62	(2, 132) 0.61	(2, 46) 0.49	(2, 132) 0.15	(2, 46) 0.23
Group × condition	(2, 132) 0.60	(2, 46) 0.55	(2, 132) 3.40^*^	(2, 46) 2.15	(2, 132) 1.77	(2, 46) 1.13	(2, 132) 1.72	(2, 46) 1.45
**FIRST PASS TIME**								
Group	(1, 66) 28.95^***^	(1, 23) 61.04^***^	(1, 66) 59.59^***^	(1, 23) 259.90^***^	(1, 66) 56.62^***^	(1, 23) 127.10^***^	(1, 66) 50.08^***^	(1, 23) 194.90^***^
Condition	(2, 132) 0.06	(2, 46) 0.34	(2, 132) 1.09	(2, 46) 1.54	(2, 132) 0.33	(2, 46) 0.57	(2, 132) 0.18	(2, 46) 0.15
Group × condition	(2, 132) 1.34	(2, 46) 0.96	(2, 132) 1.12	(2, 46) 0.57	(2, 132) 2.21	(2, 46) 1.58	(2, 132) 1.14	(2, 46) 0.83
**REGRESSION-PATH TIME**								
Group	(1, 66) 19.29^***^	(1, 23) 53.77^***^	(1, 66) 26.21^***^	(1, 23) 91.55^***^	(1, 66) 27.98^***^	(1, 23) 65.59^***^	(1, 66) 5.38^*^	(1, 23) 35.49^***^
Condition	(2, 132) 1.27	(2, 46) 1.99	(2, 132) 10.52^***^	(2, 46) 14.28^***^	(2, 132) 4.59^*^	(2, 46) 8.54^***^	(2, 132) 9.92^***^	(2, 46) 9.59^***^
Group × condition	(2, 132) 0.84	(2, 46) 0.72	(2, 132) 0.84	(2, 46) 0.41	(2, 132) 1.44	(2, 46) 1.79	(2, 132) 0.06	(2, 46) 0.13
**REREADING TIME**								
Group	(1, 66) 1.91	(1, 23) 8.14^**^	(1, 66) 0.93	(1, 23) 1.89	(1, 66) 0.21	(1, 23) 1.40	(1, 66) 0.21	(1, 23) 0.90
Condition	(2, 132) 21.73^***^	(2, 46) 23.68^***^	(2, 132) 17.38^***^	(2, 46) 19.16^***^	(2, 132) 5.45^**^	(2, 46) 2.35	(2, 132) 4.52^*^	(2, 46) 2.36
Group × condition	(2, 132) 1.62	(2, 46) 2.99(^*^)	(2, 132) 0.03	(2, 46) 0.30	(2, 132) 1.14	(2, 46) 0.52	(2, 132) 0.70	(2, 46) 0.87
**TOTAL VIEWING TIME**								
Group	(1, 66) 8.33^**^	(1, 23) 24.88^***^	(1, 66) 14.00^***^	(1, 23) 50.99^***^	(1, 66) 26.45^***^	(1, 23) 69.77^***^	(1, 66) 31.11^***^	(1, 23) 179.10^***^
Condition	(2, 132) 26.81^***^	(2, 46) 30.38^***^	(2, 132) 19.94^***^	(2, 46) 23.47^***^	(2, 132) 6.74^**^	(2, 46) 6.83^**^	(2, 132) 2.28	(2, 46) 1.72
Group × condition	(2, 132) 0.55	(2, 46) 1.60	(2, 132) 0.32	(2, 46) 0.31	(2, 132) 1.70	(2, 46) 1.72	(2, 132) 2.20	(2, 46) 1.99

(*) p < 0.1;

* p < 0.05;

** p < 0.01;

*** *p < 0.001*.

**Table 3:** **Planned pairwise comparisons between conditions for Experiment 2**.

	**Pronoun region**		**Spillover region**		**Prefinal region**		**Final region**	
	***t*1**	***t*2**	***t*1**	***t*2**	***t*1**	***t*2**	***t*1**	***t*2**
**REGRESSION-PATH TIME**								
Double match (a), local mismatch (b)			(67) 0.93	(23) 0.84	(67) 0.57	(23) 0.71	(67) 1.04	(23) 0.97
Double match (a), non-local mismatch (c)			(67) −3.23^**^	(23) −4.43^***^	(67) −2.27^*^	(23) −4.56^***^	(67) −3.09^**^	(23) −3.32^**^
Local mismatch (b), non-local mismatch (c)			(67) −4.48^***^	(23) −4.87^***^	(67) −2.66^*^	(23) −3.74^**^	(67) −4.34^***^	(23) −4.23^***^
**REREADING TIME**								
Double match (a), local mismatch (b)	(67) 1.20	(23) 0.87	(67) 0.92	(23) 0.64	(67) 0.99	(23) 0.77	(67) 0.96	(23) 0.75
Double match (a), non-local mismatch (c)	(67) −4.94^***^	(23) −4.68^***^	(67) −4.43^***^	(23) −4.73^***^	(67) −2.19^*^	(23) −1.76(^*^)	(67) −1.83(^*^)	(23) −1.53
Local mismatch, non-local mismatch	(67) −6.05^***^	(23) −6.18^***^	(67) −5.42^***^	(23) −5.73^***^	(67) −3.11^**^	(23) −2.40^*^	(67) −3.08^**^	(23) −2.03(^*^)
**TOTAL VIEWING TIME**								
Double match (a), local mismatch (b)	(67) 1.41	(23) 0.81	(67) 1.08	(23) 0.87	(67) 1.26	(23) 1.05		
Double match (a), non-local mismatch (c)	(67) −5.77^***^	(23) −5.42^***^	(67) −4.56^***^	(23) −5.59^***^	(67) −2.32^*^	(23) −2.67^*^		
Local mismatch (b), non-local mismatch (c)	(67) −6.26^***^	(23) −7.97^***^	(67) −6.02^***^	(23) −5.80^***^	(67) −3.46^**^	(23) −3.36^**^		

(*) p < 0.1;

* p < 0.05;

** p < 0.01;

*** *p < 0.001*.

First-fixation durations, first-pass times and regression-path times in the region prior to the pronoun were also examined in order to check whether any effects of condition began before the pronoun was encountered. This precritical region consisted of the word before the pronoun (excluding the final three letters, which forms part of the pronoun region), and the previous word which was always an auxiliary verb. Skipping rates in this region were 11% for the L1 group and 2% for the L2 group. No effects of Condition, or Condition by Group interactions, were found in first-pass times or regression-path times. First-fixation durations did show a main effect of Condition (marginal in the *F*_2_ analysis): [*F*_1(2, 132)_ = 3.89, *p* < 0.05; *F*_2(2, 46)_ = 2.47, *p* = 0.09.] Pairwise comparisons revealed that first-fixation durations were significantly longer in the local mismatch condition (8b) compared to the double match condition (8a) [*t*_1(67)_ = 2.79, *p* < 0.05; *t*_2(23)_ = 2.26, *p* < 0.05] and (marginally) longer than the non-local mismatch condition (8c) [*t*_1(67)_ = 1.85, *p* = 0.07; *t*_2(23)_ = 2.12, *p* < 0.05]. This effect is very fleeting, and is in a different direction from the effects seen at and beyond the pronoun region. It will therefore not be discussed any further.

### Pronoun region

Significant or partially significant main effects of Group were seen in all eye-movement measures, reflecting the fact that the L2 participants read the stimulus sentences generally more slowly than the L1 group. No main effects of, or interactions with, the factor Condition were found for first fixation durations or first-pass reading times. For both participant groups, regression path, rereading and total viewing times were longest in the non-local mismatch condition (8c), where the pronoun mismatched the accessible antecedent's gender, however. Significant main effects of Condition, unmodulated by the factor Group, were found for rereading and total viewing times. Subsequent *t*-tests on the collapsed L1 and L2 data confirmed that the pronoun region was reread significantly more slowly in the non-local mismatch condition (8c) compared to both the local mismatch (8b) and the double match condition (8a). The same statistical pattern was found for total viewing times.

### Spillover region

A similar pattern was seen at the spillover region. Main effects of Group were present in all measures other than rereading time. Both groups again showed the longest reading times in the non-local mismatch condition in regression path, rereading and total viewing times, giving rise to significant main effects of Condition unmodulated by the factor Group. Subsequent pairwise comparisons confirmed that in all three of these measures, the non-local mismatch condition elicited significantly longer reading times than the double match and local mismatch conditions.

The L2 group differed from the native readers in that the above reading-time pattern was also seen, numerically, in the L2 readers' first fixation durations and first-pass times at the spillover region. A Group by Condition interaction was found for first fixation durations that was significant by subjects only. To further explore this interaction, separate one-way ANOVAs for each group (L1 and L2) were carried out. These showed a significant effect of Condition for the L2 [*F*_1(2, 66)_ = 3.81, *p* < 0.05; *F*_2(2, 46)_ = 5.02, *p* < 0.05] but not for the L1 group [*F*_1(2, 66)_ = 0.76, *p* = 0.47; *F*_2(2, 46)_ = 0.29, *p* = 0.75]. In the L2 group first fixation durations were marginally longer, by items, in the non-local mismatch condition (8c) compared to the double match condition (8a) [*t*_1(33)_ = 1.69, *p* = 0.10; *t*_2(23)_ = 2.61, *p* < 0.05] and significantly longer compared to the local mismatch condition (8b) [*t*_1(33)_ = 2.56, *p* < 0.05; *t*_2(23)_ = 2.68, *p* < 0.05].

### Prefinal and final regions

Main effects of Group were again seen at the prefinal and final regions, alongside main effects of Condition not modulated by Group. In the prefinal region significant condition effects were found in regression path and total viewing times, with the effect significant by subjects only in rereading times. Pairwise comparisons once again revealed significant differences between the non-local mismatch condition (8c) and both the double match (8a) and the local mismatch condition (8b) for regression path, rereading and total viewing times. In the final region there was a main effect of condition in the regression-path times (also a main effect significant by subjects in rereading times). Pairwise comparisons again revealed significant differences between the non-local mismatch condition (8c) and both the double match (8a) and the local mismatch condition (8b) for regression path times, with marginal differences in the same direction for rereading times.

### Summary, experiment 2

In Experiment 2 the two participant groups patterned largely alike. Participants showed sensitivity to gender-mismatching non-local (i.e., accessible) antecedents but not to mismatching local (i.e., inaccessible) antecedents. These non-local mismatch effects were generally restricted to later reading-time measures, including total viewing times, with the exception of the L2 group's first fixation durations at the spillover region. This relatively minor between-groups difference might be due to the non-native readers' generally more “serial” reading strategy (as reflected by their lower skipping rates). Participants showed no evidence of considering the local antecedent at any point during processing, a finding that is consistent with the BAIF hypothesis.

The accessible-mismatch effects we observed are also in line with the results from the offline antecedent choice task, where both participant groups consistently chose the non-local antecedent.

The predictions of the defeasible filter hypothesis are not borne out here, because there is no evidence that either group considered the inaccessible antecedent at a later point during processing.

Note, however, that it is theoretically possible that the non-local mismatch effects seen in Experiment 2 reflect a general preference for matrix subject antecedents rather than the application of condition B. Examining the processing of SDPs should be able to confirm or rule out this hypothesis. It also allows us to see whether feature matching plays a more important role in L1 and/or L2 processing in the absence of a structural constraint which rules out one of the antecedents.

## Materials and methods, experiment 3

Our second eye-movement experiment examined the real-time processing of pronouns believed to be exempt from condition B. Recall that in the offline task (Experiment 1), both L1 and L2 participants showed awareness of the ambiguity of SDPs. However, in cases where one specific antecedent was chosen, L2s preferred the non-local antecedent whereas for L1s there was no preference. Online, will L1 and L2 participants show sensitivity to the gender of the local or non-local antecedent, or both antecedents?

### Participants

These were the same as in Experiment 2.

### Materials

The materials for this experiment included 18 experimental items which were again composed of three sentences each, a lead-in sentence, a critical sentence that contained the pronoun and two potential antecedents, and a wrap-up sentence. The gender match between the two names and the pronoun was manipulated to create three conditions as illustrated in (9a–c).

(9) Suddenly the lights went on and there were police everywhere.
  - Double match condition
    Barry saw Gavin place a gun near him on the ground with great care.
  - Local mismatch condition
    Barry saw Megan place a gun near him on the ground with great care.
  - Non-local mismatch condition
    Megan saw Barry place a gun near him on the ground with great care.
    The robbery was definitely over now.

The names were again matched in letter and syllable length, were either typical male or typical female names, and were counter-balanced across the items. Half the pronouns were masculine and half feminine. As in the materials for Experiment 2, the first name (the non-local antecedent) was always the matrix subject. The second name (the local antecedent) was always the subject of an infinitival complement of a perception verb. Unlike in Experiment 2, the pronoun here appeared inside a prepositional phrase and thus was not a coargument of the local antecedent.

### Predictions

Since SDPs are thought to be ambiguous and exempt from condition B, the predictions for Experiment 3 differ somewhat from those for Experiment 2 above.

#### Matrix-subject preference

If the parser initially searches for the matrix subject (i.e., the non-local antecedent), longer reading times are expected in the non-local mismatch condition (9c) compared to the other two conditions, similar to the results from Experiment 2.

#### Feature-match hypothesis

Where condition B does not rule out the local antecedent, the parser may be sensitive to gender mismatches between the pronoun and either or both potential antecedents. Participants might experience interference or competition when both antecedents match the pronoun in gender (condition 9a) compared to when only a single antecedent matches (conditions 9b and 9c), which would be reflected in longer reading times for the double-match condition (9a) compared to the two mismatch conditions.

Previous research on SDPs suggests that L1s are sensitive to their ambiguity in online processing tasks (Sekerina et al., 2004). For L2s there is evidence from eye-movement experiments on reflexives which indicates that they prefer linking these to the most discourse-prominent antecedent initially (Felser and Cunnings, 2012). In the light of these findings, we may expect the L2 group to show a different processing pattern from the L1 group here. While L1s might fail to show a clear antecedent preference for SDPs, or may be slowed down by antecedent competition in condition (9a), the non-native group might try to link SDPs to the matrix subject, giving rise to non-local gender mismatch effects.

### Procedures

The experimental, data cleaning and data analysis procedures for Experiment 3 were the same as in Experiment 2.

## Results, experiment 3

Responses to the comprehension questions are reported in the Results section for Experiment 2. As for Experiment 2, we will report statistical analyses for four sentence regions. The pronoun region contained the pronoun and the last three letters of the preceding preposition, the spillover region contained the two words (e.g., *on the*) immediately following the pronoun, the prefinal region two words (e.g., *ground with*) following the spillover region and the final region the final two words of the sentence. Skipping rates for these regions were 11, 20, 9, and 20% in the L1 group and 5, 4, 2, and 5% in the L2 group.

Table 4 provides an overview of the reading time data and Table 5 shows the between-groups ANOVA results of the log-transformed data in Experiment 3.

**Table 4:** **Means (standard deviations in parentheses) for five eye-movement measures at four areas of interest in Experiment 3, for each participant group**.

	**L1**					**L2**				
	**First fixation duration**	**First-pass time**	**Regression-path time**	**Rereading time**	**Total viewing time**	**First fixation duration**	**First-pass time**	**Regression-path time**	**Rereading time**	**Total viewing time**
**PRONOUN REGION**										
Double match	217 (93)	283 (164)	364 (332)	134 (223)	417 (293)	246 (85)	379 (199)	472 (490)	138 (285)	517 (353)
Local mismatch	213 (73)	287 (182)	413 (546)	154 (225)	441 (281)	244 (96)	381 (188)	442 (346)	147 (269)	528 (337)
Non-local mismatch	220 (93)	292 (169)	362 (323)	128 (203)	420 (259)	250 (99)	388 (190)	508 (697)	207 (375)	595 (415)
**SPILLOVER REGION**										
Double match	209 (74)	259 (218)	330 (324)	118 (200)	377 (288)	227 (74)	338 (166)	407 (343)	175 (341)	512 (426)
Local mismatch	201 (63)	239 (115)	350 (298)	121 (216)	361 (233)	229 (74)	353 (208)	473 (591)	148 (273)	501 (336)
Non-local mismatch	213 (73)	251 (138)	333 (358)	124 (236)	375 (278)	254 (114)	378 (222)	498 (420)	214 (368)	591 (444)
**PREFINAL REGION**										
Double match	194 (64)	293 (174)	423 (426)	123 (187)	416 (242)	225 (77)	439 (288)	542 (508)	191 (371)	630 (465)
Local mismatch	201 (80)	292 (171)	465 (616)	171 (265)	464 (304)	219 (61)	432 (253)	539 (488)	186 (341)	617 (454)
Non-local mismatch	204 (79)	296 (194)	478 (543)	146 (246)	442 (319)	215 (73)	392 (228)	539 (530)	211 (322)	603 (386)
**FINAL REGION**										
Double match	201 (96)	242 (136)	522 (629)	46 (112)	288 (168)	245 (113)	400 (249)	790 (1141)	88 (209)	487 (354)
Local mismatch	213 (89)	257 (158)	649 (1083)	77 (161)	334 (235)	233 (88)	380 (243)	751 (920)	85 (214)	465 (333)
Non-local mismatch	208 (102)	247 (140)	604 (840)	47 (112)	294 (196)	239 (127)	397 (285)	861 (1367)	132 (377)	529 (488)

**Table 5:** **Summary of analyses of variance for the pronoun, spillover, prefinal, and final region in Experiment 3**.

	**Pronoun region**		**Spillover region**		**Prefinal region**		**Final region**	
	***F*1**	***F*2**	***F*1**	***F*2**	***F*1**	***F*2**	***F*1**	***F*2**
**FIRST FIXATION DURATION**								
Group	(1, 66) 12.53^***^	(1, 17) 40.76^***^	(1, 66) 15.87^***^	(1, 17) 125.10^***^	(1, 66) 9.15^**^	(1, 17) 34.19^***^	(1, 65) 12.63^***^	(1, 17) 36.44^***^
Condition	(2, 132) 0.29	(2, 34) 0.49	(2, 132) 4.64^*^	(2, 34) 3.92^*^	(2, 132) 0.03	(2, 34) 0.02	(2, 130) 0.51	(2, 34) 0.82
Group × condition	(2, 132) 0.08	(2, 34) 0.02	(2, 132) 1.22	(2, 34) 0.98	(2, 132) 1.24	(2, 34) 1.28	(2, 130) 2.30	(2, 34) 2.29
**FIRST PASS TIME**								
Group	(1, 66) 31.01^***^	(1, 17) 136.50^***^	(1, 66) 40.99^***^	(1, 17) 98.59^***^	(1, 66) 39.68^***^	(1, 17) 111.00^***^	(1, 65) 41.26^***^	(1, 17) 88.55^***^
Condition	(2, 132) 0.32	(2, 34) 0.51	(2, 132) 1.05	(2, 34) 0.47	(2, 132) 0.43	(2, 34) 2.53(^*^)	(2, 130) 0.19	(2, 34) 0.54
Group × condition	(2, 132) 0.05	(2, 34) 0.17	(2, 132) 0.72	(2, 34) 0.63	(2, 132) 0.36	(2, 34) 1.13	(2, 130) 1.85	(2, 34) 1.49
**REGRESSION-PATH TIME**								
Group	(1, 66) 20.22^***^	(1, 17) 68.34^***^	(1, 66) 21.27^***^	(1, 17) 44.09^***^	(1, 66) 18.76^***^	(1, 17) 48.14^***^	(1, 65) 8.4^**^	(1, 17) 50.91^***^
Condition	(2, 132) 0.17	(2, 34) 0.41	(2, 132) 2.54(^*^)	(2, 34) 1.39	(2, 132) 0.17	(2, 34) 0.03	(2, 130) 0.42	(2, 34) 0.20
Group × condition	(2, 132) 0.63	(2, 34) 0.27	(2, 132) 4.44^*^	(2, 34) 1.94	(2, 132) 0.06	(2, 34) 0.09	(2, 130) 0.95	(2, 34) 0.84
**REREADING TIME**								
Group	(1, 66) 0.36	(1, 17) 1.05	(1, 66) 0.59	(1, 17) 1.62	(1, 66) 0.18	(1, 17) 0.07	(1, 65) 1.45	(1, 17) 4.51^*^
Condition	(2, 132) 2.41(^*^)	(2, 34) 2.59(^*^)	(2, 132) 0.62	(2, 34) 0.40	(2, 132) 1.18	(2, 34) 1.63	(2, 130) 0.67	(2, 34) 0.34
Group × condition	(2, 132) 1.37	(2, 34) 1.83	(2, 132) 2.93(^*^)	(2, 34) 1.92	(2, 132) 1.55	(2, 34) 2.52(^*^)	(2, 130) 3.20^*^	(2, 34) 2.67(^*^)
**TOTAL VIEWING TIME**								
Group	(1, 66) 14.93^***^	(1, 17) 34.58^***^	(1, 66) 21.65^***^	(1, 17) 74.72^***^	(1, 66) 22.55^***^	(1, 17) 56.16^***^	(1, 65) 29.61^***^	(1, 17) 102.8^***^
Condition	(2, 132) 1.52	(2, 34) 1.71	(2, 132) 2.05	(2, 34) 0.87	(2, 132) 0.33	(2, 34) 0.33	(2, 130) 0.58	(2, 34) 0.42
Group × condition	(2, 132) 1.22	(2, 34) 1.02	(2, 132) 2.79(^*^)	(2, 34) 1.79	(2, 132) 0.28	(2, 34) 1.16	(2, 130) 3.64^*^	(2, 34) 5.04^*^

(*) p < 0.1;

* p < 0.05;

** p < 0.01;

*** *p < 0.001*.

As for Experiment 2, a precritical region was examined in order to check whether any effects of condition began before the pronoun was encountered. This consisted of the preposition preceding the pronoun (excluding the final three letters) and the previous one or two words forming the object of the second verb. Skipping rates in this region were 5% for the L1 group and 1% for the L2 group, No effects of Condition, or Condition by Group interactions, were found in first-fixation durations, first-pass times or regression-path times.

### Pronoun region

At the pronoun region the native readers showed the longest regression path, rereading and total viewing times for the local mismatch condition (9b) numerically, whereas the L2 group consistently showed the longest reading times for the non-local mismatch condition (9c). No significant main effects or interactions (other than main effects of Group in all measures except rereading times) were found at this region, however.

### Spillover region

At the two words following the pronoun, main effects of Group were once again seen in all measures except rereading times. The L2 group—but not the L1 group—again showed the longest reading times in the non-local mismatch condition (9c) in all five eye-movement measures numerically. The initial omnibus ANOVA revealed a main effect of Condition in first fixation durations, as well as significant Group by Condition interaction in regression path times in the analysis by subjects. Marginal interactions, by subjects only, were also found for rereading and total viewing times. As the observed (marginal) interactions, in the presence of significant main effects of Group, are indicative of between-group differences, we went on to analyze each group's reading-time data for the spillover region separately. Whilst the L1 group showed no significant effects at this region, the L2 group showed a significant main effect of Condition for first fixation durations [*F*_1(2, 66)_ = 4.82, *p* < 0.05; *F*_2(2, 34)_ = 5.41, *p* < 0.01] and significant effects, in the analyses by subjects, for regression path [*F*_1(2, 66)_ = 5.46, *p* < 0.01; *F*_2(2, 34)_ = 2.97, *p* = 0.06] and total viewing times [*F*_1(2, 66)_ = 5.67, *p* < 0.01; *F*_2(2, 34)_ = 3.22, *p* = 0.05]. Planned pairwise comparisons showed that the non-local mismatch condition (9c) was read significantly more slowly than both the double match (9a) [*t*_1(33)_ = 3.08, *p* < 0.01; *t*_2(17)_ = 2.73, *p* < 0.05] and the local mismatch (9b) conditions [*t*_1(33)_ = 2.36, *p* < 0.05; *t*_2(17)_ = 2.57, *p* < 0.05] in first fixation durations, significantly more slowly (by subjects) than the double match condition in regression path [*t*_1(33)_ = 3.34, *p* < 0.01; *t*_2(17)_ = 2.06, *p* = 0.05] and total viewing times [*t*_1(33)_ = 3.10, *p* < 0.01; *t*_2(17)_ = 1.95, *p* = 0.07], and significantly more slowly than the local mismatch condition in total viewing times [*t*_1(33)_ = 2.87, *p* < 0.01; *t*_2(17)_ = 2.13, *p* < 0.05].

### Prefinal and final regions

No significant effects or interactions, other than main effects of Group, were found at the prefinal region. At the final sentence region, interactions between Condition and Group were observed for both rereading times (marginal by items) and total viewing times. Here the L1 group showed the longest reading times for the local mismatch condition (9b) in these measures, whereas the L2 group again had longer reading times for the non-local mismatch (9c) than for the other two conditions. Subsequent per-group analyses only yielded a marginally significant main effect of Condition for the L1 group's total viewing times [*F*_1(2, 64)_ = 2.98, *p* = 0.06; *F*_2(2, 34)_ = 2.5, *p* = 0.09], and a marginal one in the by-items analysis for the L2 group's rereading times [*F*_1(2, 66)_ = 2.46, *p* = 0.09; *F*_2(2, 34)_ = 0.85, *p* = 0.43], however.

### Correlation of reading times with OPT score and offline choices

To investigate whether, for the L2 participants, the slower reading times in the non-local mismatch condition in the spillover region (9c) originate from a lack of knowledge about SDP structures among those participants with lower OPT scores, both OPT score and offline antecedent choice rates from Experiment 1 were correlated against reading times^9^. The difference between mean total viewing time in conditions (9b) and (9c) in the spillover region was calculated per participant as a measure of an individual's processing difficulty on encountering a mismatching non-local antecedent. However, there was no significant correlation between this reading measure and either OPT score [*r*_(34)_ = −0.14, *p* = 0.4] or antecedent choice rates [*r*_(34)_ = 0.03, *p* = 0.8].

### Summary, experiment 3

In Experiment 3 we saw differences between the L1 and L2 groups' reading-time patterns, in particular in the spillover region. In the pronoun region, the trend in the L1 data was for increased reading times in the local mismatch condition (9b) while the L2 trend was for increased times in the non-local mismatch condition (9c). Although these different patterns did not yield statistically reliable between-groups differences in the pronoun region, they gave rise to some interactions with the factor Group in later regions. In the spillover region the L1s showed no significant differences between the experimental conditions whilst the L2s showed increased reading times for the non-local mismatch condition (9c), indicative of trying to link the pronoun to the matrix subject. Analysis of the L1 data in the final region revealed a trend toward longer total viewing times in the local mismatch condition (9b). In the following section, the results from Experiment 3 will be discussed together with those from Experiments 1 and 2.

## Discussion

We set out to investigate the application and timing of condition B during L1 and L2 processing of English pronouns. Firstly, we discovered that both L1 and L2 groups were sensitive to the gender of the accessible antecedent online. There was an increase in reading times when the non-local (accessible) antecedent mismatched the pronoun's gender in canonical condition B environments. Secondly, we discovered that when both antecedents were structurally available (in SDP environments), L2s were again sensitive to the gender of the non-local antecedent (which was the matrix subject) while L1s experienced some difficulty with the local mismatch condition.

### Structural sensitivity

Results from the offline questionnaire (Experiment 1) revealed that both the L1s and L2s ignored an inaccessible but gender-matching antecedent and instead chose the accessible antecedent almost exclusively, in line with condition B. This offline adherence to condition B was also reflected online in both groups, who showed longer reading times in the non-local mismatch condition in Experiment 2. This indicates a higher processing cost when the available antecedent mismatched in gender with the pronoun. No measurable processing cost was elicited by a mismatching inaccessible antecedent at any point, indicating that the inaccessible antecedent was not considered^10^. Furthermore, the results from Experiment 3 for the L1 group suggest that there may be no general preference for the first-mentioned antecedent, so it is unlikely that the Experiment 2 results were driven by such an underlying preference. These findings are line with the BAIF hypothesis, in which condition B gates access to the potential antecedents by filtering out structurally inaccessible ones. As such it adds to the evidence gained from the self-paced reading studies of Clifton et al. (1997, 1999), as well as self-paced reading and eye-tracking evidence from Chow et al. (in preparation). Because of the sensitivity of the eye-movement monitoring technique used in the current experiments, the evidence here suggests that previous support for the BAIF is not simply due to a less sensitive time measure which failed to pick up on short-lived, early effects.

The L1 data from Experiment 3 showed a trend for late processing difficulty in the local-mismatch condition, although this did not prove statistically reliable. This might nevertheless suggest that, while the native readers were largely unaffected by our manipulations of gender congruence between the pronoun and the potential antecedents, they had a weak preference for a local antecedent online. No such preference was visible in the L1 group's offline data, however. In the SDP environments both of the antecedents were accessible, and all experimental conditions contained at least one gender-matching accessible antecedent. This may explain the relative lack of any condition-specific processing difficulty in comparison to the condition B environments. The fact that the SDP items were processed differently despite being presented in same experimental session as the condition B items highlights that the L1 parser was sensitive to the subtle syntactic cues which distinguish SDP environments from those in which condition B applies.

### Timing

With respect to timing, it should first be noted that the L2 group showed sensitivity to our experimental manipulation in an earlier measure than did the L1 group in Experiment 2 (first fixation durations at the spillover region). In fact, the timing of the non-local mismatch effect in this experiment for the L1 group appears to be fairly late, appearing only in rereading times. The emergence of the L1 effect in rereading times could be due to a rapid reading strategy leading to fewer fixations and longer saccades, but increased regressive eye-movements in case of difficulty. In contrast, the L2s read more slowly, spending more time in each region. These differences in reading style might explain the seemingly earlier effects in the L2 group compared to the L1 group.

The timing of the effect in L1s, however, still stands in contrast to findings for inaccessible mismatch effects in previous (L1) studies with reflexives (e.g., Sturt, 2003). The comparison with reflexive studies is speculative because reflexives were not systematically tested in the current study. However some further consideration should be given to timing, since the study employs a method that is particularly sensitive to timecourse. It cannot be assumed that early and late reading measures are necessarily linked to distinct cognitive processes (see Pickering et al., 2004 for a discussion). As such, the effects in the rereading times could be behavioral echoes of much earlier processes. Even so, a later effect for pronouns fits in well with two considerations: first, pronouns are sensitive to a range of cues or information types which can help to determine their reference, so considering all these information sources may require more time; second, the nature of condition B, unlike condition A for reflexives, involves excluding rather than identifying an antecedent, and may require the generation of more than one semantic sentence representation (Reuland, 2001, 2011) or the consideration of pragmatic information (Huang, 1994).

### L1 vs. L2 processing

The L2 group showed a very similar pattern of results to the L1 group in Experiment 2, but a different pattern of results from the L1 group in Experiment 3. Although the results of Experiment 2 suggest that L2s do rule out the inaccessible antecedent in accordance with condition B (like the L1 group), results from Experiment 3 for the L2 group call this into question. In Experiment 3, the L2 participants were again sensitive to the gender of the non-local antecedent, despite their awareness of the ambiguity in the offline task (Experiment 1). This means that their sensitivity to the non-local antecedent in Experiment 2 may not be a result of applying condition B, but could instead be a general preference to link the pronoun to the matrix subject, even though offline the L2s show awareness of the ambiguity of the SDPs. This suggests firstly that L2s are less sensitive than L1s to the subtle syntactic cues that differentiate the SDP environments from the canonical condition B environments. Secondly, they appear to have a general preference for salient subjects, which may have driven the non-local mismatch effect for L2s in both Experiments 2 and 3. The discrepancy between L2s' offline knowledge and their use of this knowledge during online processing has been observed in previous studies, as well as a preference for (discourse-) salient antecedents (Felser and Cunnings, 2012 for reflexives). This finding is consistent with the hypothesis that L2 speakers tend to underuse structural information during processing and rely more on other cues such as discourse-level information instead (Clahsen and Felser, 2006).

A reviewer raises the question of whether the German participants' preference for non-local antecedents in Experiment 3 might reflect L1 transfer. Similar SDP configurations to those tested here also exist in German. To find out which, if any, antecedent native German readers might prefer online, we carried out a parallel eye-movement study on German (as yet unpublished). While L1 German readers showed an offline preference for the non-local antecedent, their reading-time patterns look similar to those of the native English group in the current study in that they did not show any measurable preference for either the local or non-local antecedent. The double-match condition tended to be the shortest one instead, a pattern that proved statistically significant only for total viewing times at the spillover region, however. This makes it unlikely that our Experiment 3 results reflect L1 transfer from German^11^.

### Implications for antecedent search mechanisms

The predictions of the BAIF hypothesis for pronouns appear to be very similar to those of a structured search mechanism for reflexives (Dillon, 2011; Dillon et al., 2013). If readers show sensitivity to the conditions governing both reflexives and pronouns, can they be assumed to exploit the same search mechanism? This makes the assumption that condition B is purely a structural constraint, a proposal which is contested by several theoretical accounts. A purely structured search to eliminate an inaccessible antecedent may therefore be inadequate. Nevertheless, a model of memory search for pronouns must incorporate (i) the ability to exclude an inaccessible antecedent from consideration even when it carries features that match the pronoun, and (ii) awareness of explicitly structural cues that distinguish, for example, canonical condition B environments from SDP environments. It is clear that native speakers make use of this information during processing, and that it plays a decisive role during the consideration of potential antecedents.

A slightly different question is whether there is a strict ordering of constraint application, as Nicol and Swinney imply in their original formulation of their hypothesis:

“… the reactivation of prior referents is restricted by grammatical constraints. In the case where such information does not sufficiently constrain the list of potential antecedents to a single one, the pragmatic and other sentence/discourse processing procedures undoubtedly come into play, but, given the present evidence, only at a later point in processing.”

(Nicol and Swinney, 1989, p.18)

While the lack of interference from an inaccessible antecedent seems to imply that binding conditions are applied before other cues such as gender features are recruited, there is as yet no firm evidence that discourse cues, for example, are systematically withheld relative to binding constraints in the time-course of pronoun resolution. Given that discourse cues are increasingly found to act early and even predictively (e.g., Koornneef and Van Berkum, 2006; Cozjin et al., 2011), further research on the interaction between condition B and the discourse status of antecedents would be welcome, to confirm or disconfirm a strict ordering of constraint application.

In addition, any model of the retrieval process should be able to incorporate the profiles of both native and non-native comprehenders. As far as the L2 processing is concerned, the current study shows that the processing of pronouns may be driven by a search for a salient subject, rather than making use of a detailed structural analysis to distinguish condition B and SDP environments; this is not the case for L1 processing. This demonstrates a different sensitivity to structural cues in the two populations; generalizing a retrieval or processing model so that it applies equally well to L1 and L2 pronoun resolution could perhaps be achieved by assigning differing constraint weights in different populations.

## Conclusion

Native English speakers appear to successfully apply condition B online so that they do not consider an inaccessible antecedent at any point during processing, which is in line with the BAIF hypothesis. They are also sensitive to syntactic cues that distinguish syntactic environments that either require, or do not require, the exclusion of a local referent. By contrast, non-native speakers do not appear to distinguish condition B environments from SDP environments online, appearing to opt for salient subject antecedents in both despite offline awareness of the difference. The different processing profiles of native and non-native speakers must be incorporated into models of retrieval, with particular reference to the relative importance of structural cues for different populations.

### Conflict of interest statement

The authors declare that the research was conducted in the absence of any commercial or financial relationships that could be construed as a potential conflict of interest.

## Footnotes

^1^For simplicity, reflexive pronouns will henceforth be referred to as reflexives and non-reflexive pronouns will be referred to as pronouns.

^2^The term “c-command” refers to a particular structural relationship between constituents that is defined in terms of hierarchical dominance (Reinhart, 1983). If, for example, a pronoun is contained within a category that is dominated by the same branching node that immediately dominates another NP, that NP is said to c-command the pronoun.

^3^A typical example of a picture noun phrase is “Nick's picture of himself/him,” where both the reflexive and the pronoun can be understood as referring to Nick.

^4^The reason for the larger number of participants in the L1 group compared to the L2 group is discussed in the “procedure” section.

^5^Additionally, responses of both L1 and L2 participants to unambiguous filler items were checked to ensure that the participants had understood the task. The percentage of correct answers was 98% for the L1 and 93% for the L2 group.

^6^We remain cautious about this observation, firstly because of the limited range of the OPT scores, and secondly because the OPT gives placement scores (sufficient to demonstrate that all L2 participants were competent in English), rather than a direct and thorough measure of proficiency. Additionally, we did not set out to test the effect of proficiency here, and have made no specific predictions.

^7^A potential fourth condition in which neither name matched the pronoun in gender was not included in order to avoid presenting participants with too many unresolvable pronouns, which could have drawn their attention to the pronouns and encouraged strategic reading behavior. This is also the case for the materials of Experiment 3.

^8^Trials for which (L2) participants had indicated unknown vocabulary were not removed from the analysis reported here. A parallel analysis with unknown vocabulary trials excluded showed that excluding these did not affect the results.

^9^We thank the reviewers for this suggestion.

^10^However, it should be noted that a previous analysis of the Experiment 2 data, in which the pronoun region contained only the pronoun itself, the L2 group did appear to be briefly distracted by a gender-matching, inaccessible antecedent. Following a reviewer's suggestion, this analysis was replaced due to high skipping rates and the resultant loss of data.

^11^Note that in order to draw any meaningful conclusions about possible L1 transfer, learner groups from different L1 backgrounds would need to be compared.
